# Depression and risk of infectious diseases: A mendelian randomization study

**DOI:** 10.1038/s41398-024-02950-8

**Published:** 2024-06-08

**Authors:** Luchen Shi, Junsong Ren, Ke Jin, Jun Li

**Affiliations:** 1 Department of Infectious Diseases, The First Affiliated Hospital of Nanjing Medical University, Nanjing, Jiangsu China; 2 The Affiliated Eye Hospital, Nanjing Medical University, Nanjing, Jiangsu China

**Keywords:** Depression, Clinical genetics

## Abstract

Previous observational inquiries have revealed a correlation between depression and infectious maladies. This study seeks to elucidate the causal linkages between depression, specifically Major Depressive Disorder (MDD), and infectious diseases. Nevertheless, the causative nature of the association between MDD and infectious diseases remains elusive. Two-sample Mendelian Randomization (MR) analyses was executed utilizing single nucleotide polymorphisms (SNPs) significantly connected with MDD and infectious diseases as instrumental variables (IVs). A series of sensitivity analyses were subsequently conducted. Genetic variants linked to MDD were employed as instrumental variables sourced from a genome-wide meta-analyses comprising 500,199 individuals. Summary-level data on five infectious diseases, including candidiasis, pneumonia, skin and soft tissue infections (SSTI), upper respiratory tract infections (URTI), and urinary tract infections (UTI), were acquired from the UK Biobank and FinnGen study. Our findings evinced that genetically predicted MDD exhibited a heightened risk of candidiasis (OR = 1.52, 95% CI 1.06–2.17; *P* = 2.38E-02), pneumonia (OR = 1.14, 95% CI 1.01–1.29; *P* = 3.16E-02), URTI (OR = 1.23, 95% CI 1.12–1.36; *P* = 3.71E-05), and UTI (OR = 1.26, 95% CI 1.12–1.42; *P* = 8.90E-05). Additionally, we identified bidirectional causal relationships between UTI and MDD. The associations between MDD and the risk of URTI and UTI remained consistent in multivariable MR analyses, accounting for genetically predicted smoking and body mass index. In conclusion, this investigation ascertained a causal connection between MDD and the susceptibility to infectious diseases, particularly URTI and UTI.

## Introduction

MDD is recognized as one of the most common and pressing mental health problems [1]. It affects around 4.4% of people worldwide and has a high rate of recurrence [2]. MDD has been discerned to be prospectively linked to obesity, cardiac ailments, diabetes, and Systemic Lupus Erythematosus [3–5]. Nevertheless, the implications of MDD on the susceptibility to infections currently remain enigmatic.

Infection is a major global public health problem, affecting approximately one-fourteenth of the global population [6]. Recent investigations have gradually unveiled associations between MDD and the vulnerability to infectious diseases [7]. For instance, a prospective population-based study encompassing 976,398 individuals reported that depression may confer an increased risk of severe infections [8]. Likewise, a comprehensive retrospective study on US college students indicated that depression exhibited an elevated risk concerning numerous acute infectious diseases [9]. However, the causality of the association between MDD and infectious diseases remains unknown, because of unmeasured confounding and reverse causality in these observational studies.

The establishment of causality is pivotal, as it can inform clinical interventions and shape public health policy. Mendelian randomization is a method used to assess the causal relationship between exposures and outcomes [10]. This approach uses genetic variants as proxies that associate with the exposure to evaluate the causality between exposure and outcome [11]. Leveraging the design rationale of Mendelian randomization effectively overcomes the challenges presented by unmeasured confounding and reverse causation, inherent in traditional observational studies [12].

In our study, a two-sample MR was performed to investigate the potential casual effects between depression and risk of following five common infectious diseases: candidiasis, pneumonia, SSTI, URTI, UTI.

## Methods

### Study design

**Fig. 1:**
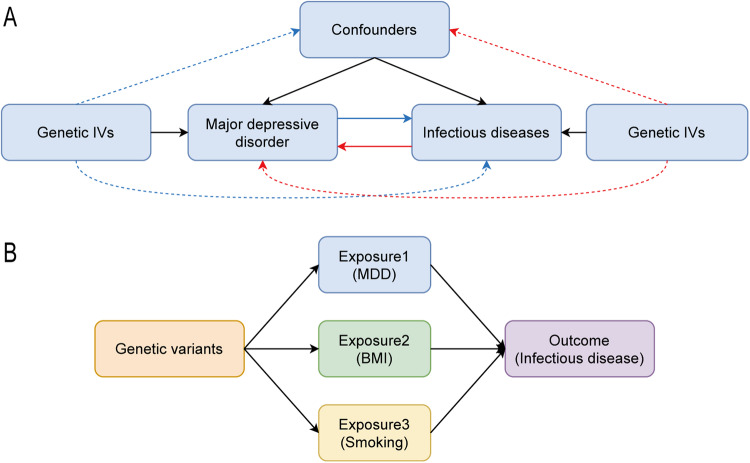
Study design. **A** Overview of this bidirectional MR study design. **B** Overview of multivariable MR study design. BMI body mass index, IVs instrumental variable, MDD major depressive disorder.

We employed a two-sample Mendelian Randomization (MR) analyses, utilizing publicly available Genome-Wide Association Study (GWAS) data on Major Depressive Disorder (MDD) and infectious diseases, to assess the causal relationships. The execution of this study adhered diligently to the fundamental principles set forth by the Strengthening the Reporting of Observational Studies in Epidemiology Using Mendelian Randomization (STROBE-MR) guidelines [13]. This study adhered to the following rules: (1) Instrumental variables were strongly associated with exposure; (2) Instrumental variables were not associated with confounders of exposure and outcome; (3) Instrumental variables affect outcome only by exposure [14]. Both the exposure and outcome cohorts were drawn exclusively from subjects of European ancestry to minimize the influence of population structure bias. A comprehensive overview of the research design is visually depicted in Fig. 1.

### Data source and selection of instrumental variables

First, we extracted single nucleotide polymorphisms (SNPs) that were strongly associated with exposure with *P* < 5 × 10^–8^. Since linkage disequilibrium (LD) can produce serious biases in causal inference, we performed the clumping procedure with R2 < 0.001 and a clumping window of > 10,000 kb based on the 1000 Genomes European reference panel. Finally, we harmonized the exposure and outcome to eliminate palindromic SNPs. In cases where traits lacked an adequate number of SNPs, we applied a more relaxed instrument threshold of P < 1×10^–5^ to ensure an adequate SNP count. Additionally, to safeguard against any influence of weak instrumental biases on causal inference, we gauged the strength of the genetic instruments for all remaining SNPs, calculating the F statistic as β^2^/se^2^. An F statistic greater than 10 is deemed a robust instrumental variable and is considered appropriate for use in MR studies [15]. The selected SNPs were utilized for subsequent analyses. A summary of these Genome-Wide Association Studies (GWAS) can be found in Table 1.

**Table 1:** Characteristics of the data used in this study.

Trait	Data source	Year	Population	Gender	Sample size	N cases	N controls
**Exposures**							
MDD	PGC	2019	European	Males and females	500199	170756	329443
**Outcomes**							
Candidiasis	FinnGen	2021	European	Males and females	216831	2015	214816
Pneumonia	FinnGen	2021	European	Males and females	218792	27370	191422
SSTI	FinnGen	2021	European	Males and females	218792	10343	208449
URTI	FinnGen	2021	European	Males and females	218792	35847	182945
UTI	UKBiobank	2021	European	Males and females	486214	21958	464256
UTI(repeated analyses)	FinnGen	2021	European	Males and females	215644	23424	192220
**Confounders**							
BMI	GIANT	2015	European	Males and females	339224		
Smoking	GSCAN	2019	European	Males and females	607291		

*PGC* Psychiatric Genomics Consortium, *GIANT* Genetic Investigation of Anthropometric Traits, *GSCAN* GWAS and Sequencing Consortium of Alcohol and Nicotine use consortium, *SSTI* Skin and soft tissue infection, *URTI* Upper respiratory tract infections, *UTI* Urinary tract infections.

Summary statistics for Major Depressive Disorder (MDD) were acquired from the most extensive genome-wide association studies dedicated to depression, encompassing a total of 500,199 individuals, consisting of 170,756 cases and 329,443 controls [16]. The genome-wide association data originated from diverse cohorts, including PGC, UK Biobank, and 23andMe. Enrolled cases are diagnosed through structured diagnostic tools that are assessed by trained interviewers, clinician-administered checklists, or chart reviews, which are required to meet DSM-IV, ICD-9, or ICD-10 standards. In total, 50 SNPs associated with depression were identified (P < 5 × 10^−8^) and without linkage disequilibrium. The details of the IVs are displayed in Supplementary Table 1.

GWAS summary data for infectious diseases were collected from the IEU Open GWAS database(https://gwas.mrcieu.ac.uk/). Summary-level GWAS data on candidiasis [finn-b-AB1_CANDIDIASIS, *N* = 216831 (including 2015 cases and 214816 controls)], pneumonia [finn-b-J10_PNEUMONIA, *N* = 218792(including 27370 cases and 191422 controls)], SSTI [finn-b-L12_INFECT_SKIN, *N* = 218792(including 10343 cases and 208449 controls)], URTI [finn-b-J10_UPPERINFEC, *N* = 218792(including 35847 cases and 182945 controls)] were from the FinnGen biobank [17] and summary-level GWAS data on UTI [ieu-b-5065, *N* = 486,214 (including 21,958 cases and 464,256 control subjects)] were published in the UK Biobank [18]. In the UK Biobank and FinnGen datasets, cases and controls are defined based on the International Classification of Diseases codes, 10th edition, extracted from hospital records. Considering that the genetic associations for UTI were extracted from UK biobank, and because traditional MR methods may be prone to bias when using overlapping populations, to validate our findings, we performed repeated analyses using genetic data on depression from FinnGen consortium database (23,424 cases and 192,220 controls) [19]. In light of the limited number of variants utilized in reverse Mendelian randomization studies and repeated analyses, we identified relevant genetic variations through the aforementioned identical procedures, employing a relatively permissive threshold. The summary information of IVs was showed in Supplementary Tables 2–7. Genetic data on smoking and BMI downloaded from the GWAS and Sequencing Consortium of Alcohol and Nicotine use consortium (GSCAN) and the Genetic Investigation of Anthropometric Traits (GIANT) respectively for subsequent multivariable MR analyses [20, 21]. As covariates BMI and smoking originated from a joint consortium that included the UK Biobank sample, in repeated analyses, I selected genetic data related to smoking (GWAS identifier: finn-b-SMOKING) and BMI (GWAS identifier: ebi-a-GCST90029007) from IEU OpenGWAS, specifically excluding the UK Biobank sample.

### Statistical analyses

To assess the causal relationship between MDD and risk of infectious disease, we applied multiple complementary approaches, including the inverse variance weighted (IVW), the MR-Egger regression, the Weighted Median, the Simple Mode and the Weighted Mode methods. The IVW method was used as the major analyses method, because it could provide the most precise results when there is no horizontal pleiotropy [22]. Additionally, through the PhenoScanner search, associations of the instrumental variables with obesity traits were identified. Given that smoking is widely recognized as a modifiable risk factor for infectious diseases, we conducted a multivariate MR analyses to account for the influence of smoking and Body Mass Index (BMI) while evaluating the direct causal impact of MDD on the risk of infectious diseases. The identical set of instrumental variables employed in the univariable MR analyses was used in the multivariable MR analyses.

Then, we carried out a series of sensitivity analyses. The Cochran’s Q test was used to detect heterogeneity in the SNP effects of the instruments. The presence of heterogeneity would be indicated if the P-value of the Cochran Q-test fell below 0.05 [23]. To further probe potential horizontal pleiotropy, we employed the MR-Egger intercept test. The MR-PRESSO method is designed to identify SNP outliers with pleiotropic effects, and subsequently, it offers an estimate that becomes consistent with the one obtained through the IVW (Inverse Variance Weighted) method after these outliers are excluded [24]. The leave-one-out sensitivity method was used to assess the effect of individual SNPs on causality [25]. We also performed the MR-Steiger directionality test to infer the direction of causality [26]. MVMR-IVW was used as the primary analyses and the MVMR-Egger method was used for sensitivity analyses in multivariable MR analyses.

Our two-sample MR analyses was conducted using R software (version 4.1.1). The packages ‘TwoSampleMR’, ‘Mendelianrandomization’, ‘MVMR’ and ‘forestploter’ were used for statistical analyses, data output and visualization. All presented P-values were two tailed, and P-values less than 0.01(0.05/5) were considered statistically significant after the Bonferroni correction. P values between 0.01 and 0.05 were deemed suggestively causal inference. In our present analyses, the MR results were presented as odds ratios (ORs) along with their corresponding 95% confidence intervals (CIs). These ORs signify the risk associated with the outcome for every unit change in exposure.

## Results

### Univariate MR analyses

#### MDD effect on infectious diseases

**Fig. 2:**
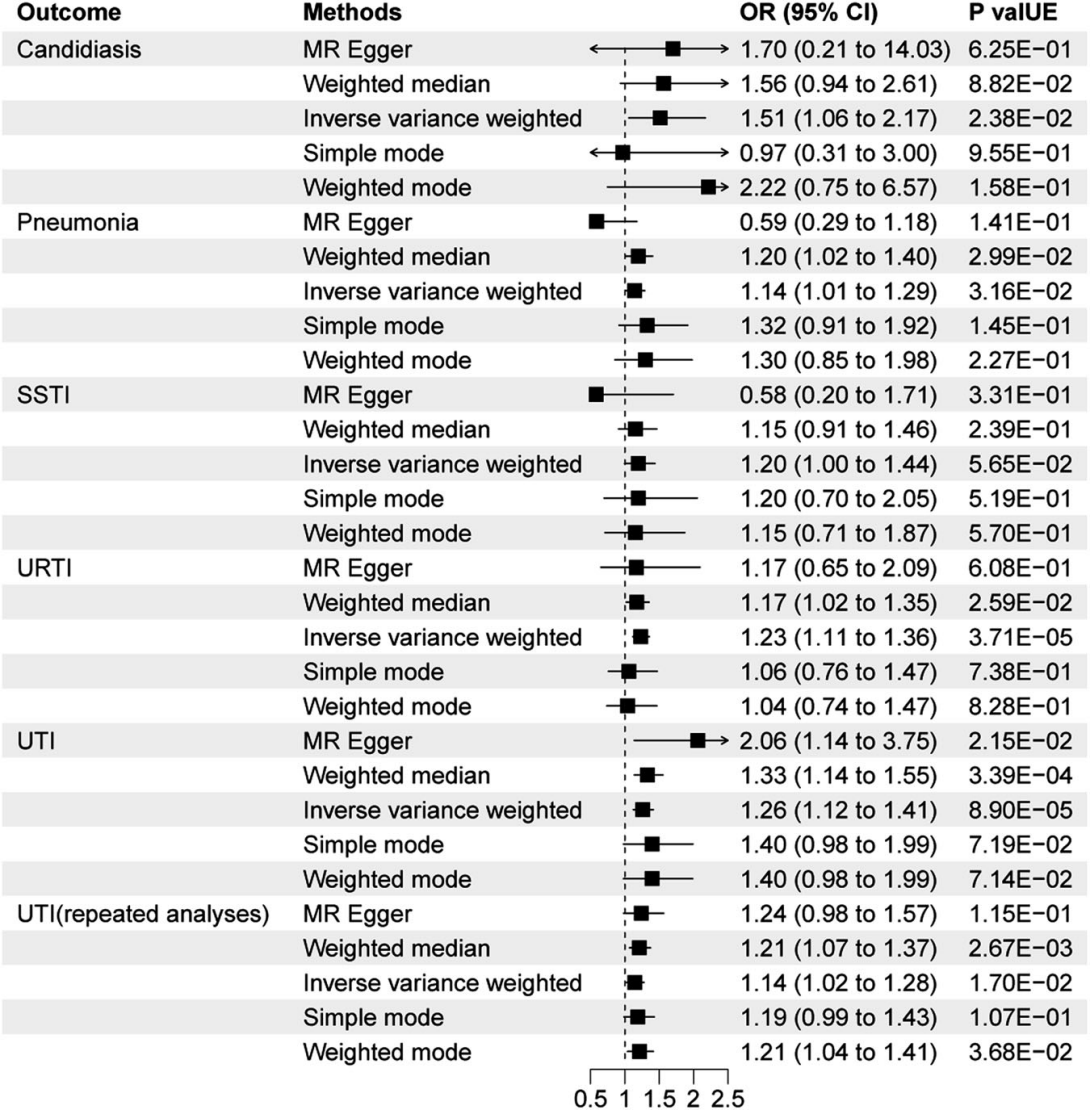
Associations of genetic liability to MDD with infectious diseases. CI confidence interval, OR odds ratio, SSTI skin and soft tissue infections, URTI upper respiratory tract infections and UTI urinary tract infections.

As shown in Fig. 2, genetically predicted MDD was associated with higher risk of candidiasis (OR = 1.52, 95% CI 1.06–2.17; P = 2.38E-02), pneumonia (OR = 1.14, 95% CI 1.01–1.29; P = 3.16E-02), URTI (OR = 1.23, 95% CI 1.12–1.36; P = 3.71E-05), and UTI (OR = 1.26, 95% CI 1.12–1.42; P = 8.90E-05). Figure S1 shows the scatter plots of univariable MR. The primary analyses was also supported by repeated analyses using MDD data from FinnGen.

In MR-Egger intercept test, there was no significant horizontal pleiotropy in all analyses (all På 0.05) (Table S8). Heterogeneity was nor detected in the Cochran’s Q analyses. In addition, no outlier was detected by MR-PRESSO approach (Table S9). The MR‐Steiger directionality test suggested that there is no inverse directionality in this study (all P < 0.05). The leave‐one‐out analyses indicated that the causal estimates of MDD and the risk of infectious diseases were not driven by any single SNP (Figure S2).

#### Infectious diseases effect on MDD

**Fig. 3:**
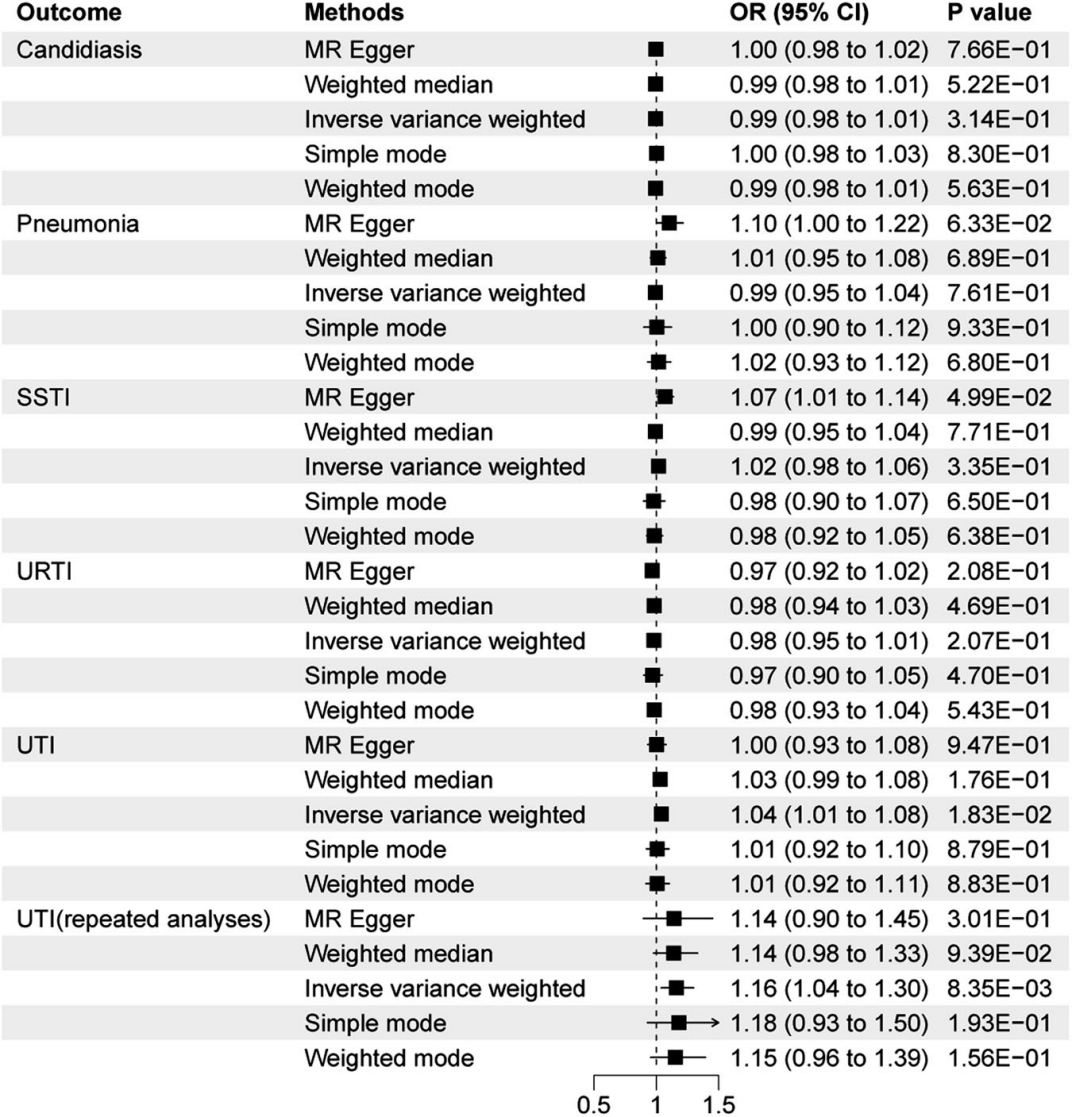
Associations of genetic liability to infectious diseases with MDD. CI confidence interval, OR odds ratio, SSTI skin and soft tissue infections, URTI upper respiratory tract infections and UTI urinary tract infections.

With genetic variants for different types of infectious diseases as exposures, MR reveals causal relationship between UTI and MDD (OR = 1.04, 95% CI 1.01–1.08; P = 1.83E-02). In repeated analyses without sample overlap, the causal relationship remains statistically significant (Fig. 3). Figure S3 shows the scatter plots of univariable MR. Results were not statistically significant for the effect of the rest infectious diseases on MDD. Horizontal pleiotropy was detected by MR-Egger regression analyses in analyses between pneumonia and MDD (Egger intercept = -0.008, P = 0.031). However, no horizontal pleiotropy was detected by the MR-PRESSO analyses(all P å 0.05) (Table S9). Heterogeneity was not detected in the Cochran’s Q analyses (Table S10). Leave-one-out analyses showed that no single SNP drove these results (Figure S4).

### Multivariable MR analyses

**Fig. 4:**
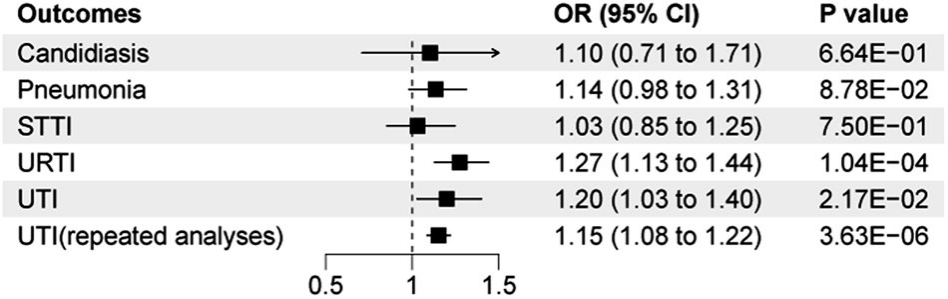
The direct effect of genetically determined MDD on infectious diseases using MVMR controlled for BMI and smoking. BMI body mass index, CI confidence interval, OR odds ratio, SSTI skin and soft tissue infections, URTI upper respiratory tract infections and UTI urinary tract infections.

The PhenoScanner search detected associations of IVs with obesity traits. Considering that smoking is also a common confounding factor, we therefore used multivariate MR analyses to adjust for smoking and BMI to assess the independent causal effects of MDD and risk for infectious diseases. The results of the multivariate MR analyses after adjusting for smoking and BMI are shown in Fig. 4. MVMR analyses identified that, for URTI, after adjusting for smoking and BMI (OR = 1.27, 95% CI: 1.13–1.44, *P* = 1.04E-04), the association remained stable. After adjusting for smoking and BMI (OR = 1.20, 95% CI: 1.03–1.40, *P* = 2.17E-02), MDD remained causally related to UTI risk. However, after adjusting for smoking and BMI, the association between MDD and risk of candidiasis, pneumonia and SSTI was not significant (på 0.05). The repeated analyses obtained similar results to the initial analyses (OR = 1.15, 95% CI: 1.08–1.22, *P* = 3.63E-06), which demonstrates the stability of the results. The MVMR-Egger methods provided consistent results and show no evidence of horizontal pleiotropy in MVMR (Table S11). Analyses on heterogeneity for the MVMR between MDD and risk of infectious diseases are shown in Table S11.

## Discussion

Utilizing publicly available summary statistics from GWAS, we performed a comprehensive two-sample MR investigation to explore the causal association between MDD and the susceptibility to infectious diseases. Our study demonstrated the causal relationship between MDD and UTI and URTI independent of BMI and smoking. Furthermore, employing bidirectional MR, we ascertained that UTI patients may face an elevated risk of MDD. To ensure the robustness of our conclusions, a series of sensitivity analyses were meticulously conducted, further consolidating the validity of our findings.

The mortality rate among individuals with mental disorders is reported to be 2.22 times higher than that of the general population [27], and infections stand as one of the leading causes of increased mortality in individuals with mental illnesses [28]. The growing attention towards the connection between mental health issues and infections is evident in recent research. A recent study revealed that loneliness is linked to an elevated risk of incident hospital-treated infections [29]. Moreover, psychological distress has been identified as a risk factor for hospitalization in SARS-CoV-2 patients [30]. A national study conducted in Korea indicated that individuals with depression have a 2.63 times higher risk of contracting tuberculosis than the general population, with a higher risk observed in those experiencing more severe depression [31]. Previous observational studies have suggested that MDD may be associated with an increased incidence of infectious diseases. In addition, a recent register-based study found a significant dose-response relationship between the number of severe infections and the risk of schizophrenia [32]. However, because of flaws in research methodology, traditional epidemiology cannot infer a causal relationship. Randomized controlled trials (RCTs) are the best method for causal inference, but the use of RCTs to assess the impact of MDD on the risk of infection is not feasible given ethical issues and funding constraints. MR has some similarities to RCTs, and it significantly reduces the effects of environmental confounders and reverse causality, we therefore chose MR to infer causality. Our findings are largely consistent with previous observational studies, which makes this finding more reliable.

The potential mechanisms between MDD and infectious diseases are not fully understood. There are several possible reasons that could explain these causal relationships. First, studies have shown that serum levels of pro-inflammatory cytokines are significantly higher in MDD patients than in normal controls [33, 34]. Infections may lead to excessive inflammatory response. We speculated that these pro-inflammatory cytokines may mediate the association of MDD with infectious diseases. Second, MDD affects the adaptive immune system, leading to increased apoptosis of CD4 + T cells and reduced T cell activity, which increases susceptibility to infectious agents [35–37]. Other possible explanation for the relationship between MDD and risk of infectious disease may be neuroendocrine dysfunction [38, 39]. The intricate interplay between the neuroendocrine system and the immune system may contribute to the heightened vulnerability to infections observed in individuals with MDD. These potential mechanisms offer valuable insights into the complex pathways linking MDD with infectious diseases.

Our findings have some implications for public health policy. If a causal relationship between depression and infections is established, it highlights the need for psychologists and healthcare professionals to be more vigilant in screening and preventing infectious diseases in individuals with depression. Conversely, if infections are found to causally influence the onset of depression, designing targeted interventions to prevent and treat infections becomes crucial in mitigating the risk of developing depression. Preventive measures for infections could potentially reduce the burden of depression in susceptible individuals. Moreover, given the bidirectional relationship observed between depression and infections, the identification of common risk factors warrants attention. Designing comprehensive interventions that address shared risk factors for both conditions could lead to significant improvements in patients’ quality of life and overall health outcomes.

This is the first MR study to investigate the causal relationship between MDD and risk of infectious diseases. Our research has several strengths. The main advantage is the rigorous MR design, which allows our study to minimize the effects of confounding factors and reverse causality. The instrumental variables we chose were closely related to the exposure of interest and were not in LD. In our analyses, the F-statistic for the instrumental variables were all well over 10, indicating the strength of the genetic instruments. Horizontal pleiotropy is a significant challenge for MR, in that genetic variation affects outcomes through other pathways than the exposure of interest. We performed multiple sensitivity analyses to minimize heterogeneity and pleiotropy. The MR-Egger intercept test showed no horizontal pleiotropy in our analyses, suggesting that our results are reliable. We additionally applied the MVMR method, adjusted for BMI and smoking, to assess the direct causal effect of MDD on risk of infectious diseases. There continued to be a strong causal link after correcting for the individual effects of BMI and smoking separately. Moreover, the associations remained stable in the repeated analyses using the genetic associations with exposures from FinnGen consortium database. All of these indications affirm the credibility of our results. The congruence of outcomes in these two cohorts further substantiates our findings. Finally, the sample size is large enough to allow us to obtain more accurate results.

Nevertheless, our study has certain limitations. Primarily, the findings are derived from genome-wide association study (GWAS) data focusing on individuals of European ancestry, raising uncertainty about generalizing these results to other ethnic groups. Although data on MDD and infectious diseases in other ethnic populations are scarce, further validation in diverse cohorts would be invaluable. Second, despite our examination of potential confounders such as smoking and body mass index (BMI) using multivariate Mendelian randomization, we were unable to fully exclude the influence of other confounding factors on the results. For instance, countries characterized by moderate to low income profiles face an aggravated burden of infectious diseases, a critical covariate that remains unassessed in the context of this study. Furthermore, it is worth noting that while certain results attained statistical significance when UTI was utilized as an exposure, the effect sizes observed were relatively modest. As such, caution is warranted in interpreting these findings, and additional independent studies are imperative to corroborate and validate the observed associations. Finally, further research is needed to explore the specific mechanisms involved in the causal effects.

In conclusion, our comprehensive MR study establishes MDD as a significant risk factor for infectious diseases. The findings highlight the potential bidirectional relationship between MDD and infections, emphasizing the importance of addressing mental health issues in the context of infectious disease prevention and management.

## Conclusion

In conclusion, this study found a causal relationship between MDD and risk of infectious diseases. We found that MDD increased the risk of UTI and URTI, while UTI in turn increased the risk of MDD. These findings reveal the intricate interplay between mental health and infectious diseases and emphasize the importance of addressing both in clinical interventions and public health strategies.

### Supplementary information

supplementary legends

Figure S1

Figure S2

Figure S3

Figure S4

Table S1

Table S2

Table S3

Table S4

Table S5

Table S6

Table S7

Table S8

Table S9

Table S10

Table S11

## Data Availability

All data analyzed in this study can be obtained by a reasonable request to corresponding authors.

## Code availability

Codes used in the current study are available from the corresponding author upon reasonable request.
