# Supplementary material for: Depression and risk of infectious diseases: A mendelian randomization study
Source: Transl Psychiatry. 2024 Jun 8;14:245. doi: 10.1038/s41398-024-02950-8 (PMC11162453; doi:10.1038/s41398-024-02950-8)
Supplement: Supplementary file 1 — supplementary legends [file 41398_2024_2950_MOESM1_ESM.docx]

Fig. S1: The scatter plot of SNP effects between MDD and infectious diseases.

Fig. S2: Leave-one-out analyses for SNPs associated with infectious diseases on MDD.

Fig. S3: The scatter plot of SNP effects between infectious diseases and MDD.

Fig. S4: Leave-one-out analyses for SNPs associated with MDD on infectious diseases.

Table S1: Instrumental variables for MDD (significant level of P < 5e-08). SNP, single nucleotide polymorphism; EAF, effect allele frequency; SE, standard error.

Table S2: Instrumental variables for Candidiasis (significant level of P < 1e-05). Palindromic SNPs that cause ambiguity were removed from further analysis. EAF: effect allele frequency; SE: standard error; ﻿SNP: single nucleotide polymorphism; MDD: major depressive disorder.

Table S3: Instrumental variables for pneumonia (significant level of P < 1e-05). Palindromic SNPs that cause ambiguity were removed from further analysis. EAF: effect allele frequency; SE: standard error; ﻿SNP: single nucleotide polymorphism; MDD: major depressive disorder.

Table S4: Instrumental variables for SSTI (significant level of P < 1e-05). Palindromic SNPs that cause ambiguity were removed from further analysis. EAF: effect allele frequency; SE: standard error; ﻿SNP: single nucleotide polymorphism; MDD: major depressive disorder.

Table S5: Instrumental variables for URTI (significant level of P < 1e-05). Palindromic SNPs that cause ambiguity were removed from further analysis. EAF: effect allele frequency; SE: standard error; ﻿SNP: single nucleotide polymorphism; MDD: major depressive disorder.

Table S6: Instrumental variables for UTI (significant level of P < 1e-05). Palindromic SNPs that cause ambiguity were removed from further analysis. EAF: effect allele frequency; SE: standard error; ﻿SNP: single nucleotide polymorphism; MDD: major depressive disorder.

Table S7: Instrumental variables for MDD (repeated analyses) (significant level of P < 1e-06). SNP, single nucleotide polymorphism; EAF, effect allele frequency; SE, standard error.

Table S8: Results of heterogeneity and pleiotropy tests. SSTI, Skin and soft tissue infections; URTI, Upper respiratory tract infections; UTI, Urinary tract infections; SE, standard error.

Table S9: Results of MR-PRESSO. CI, confidence interval; OR, odds ratio; SSTI, Skin and soft tissue infections; URTI, Upper respiratory tract infections; UTI, Urinary tract infections.

Table S10: Results of heterogeneity and pleiotropy tests. SSTI, Skin and soft tissue infections; URTI, Upper respiratory tract infections; UTI, Urinary tract infections; SE, standard error.

Table S11: Results of multivariable MR analysis with adjustment for BMI and smoking. SSTI, Skin and soft tissue infections; URTI, Upper respiratory tract infections; UTI, Urinary tract infections.
