# Supplementary figures and images for: Depression and risk of infectious diseases: A mendelian randomization study
Source: Transl Psychiatry. 2024 Jun 8;14:245. doi: 10.1038/s41398-024-02950-8 (PMC11162453; doi:10.1038/s41398-024-02950-8)

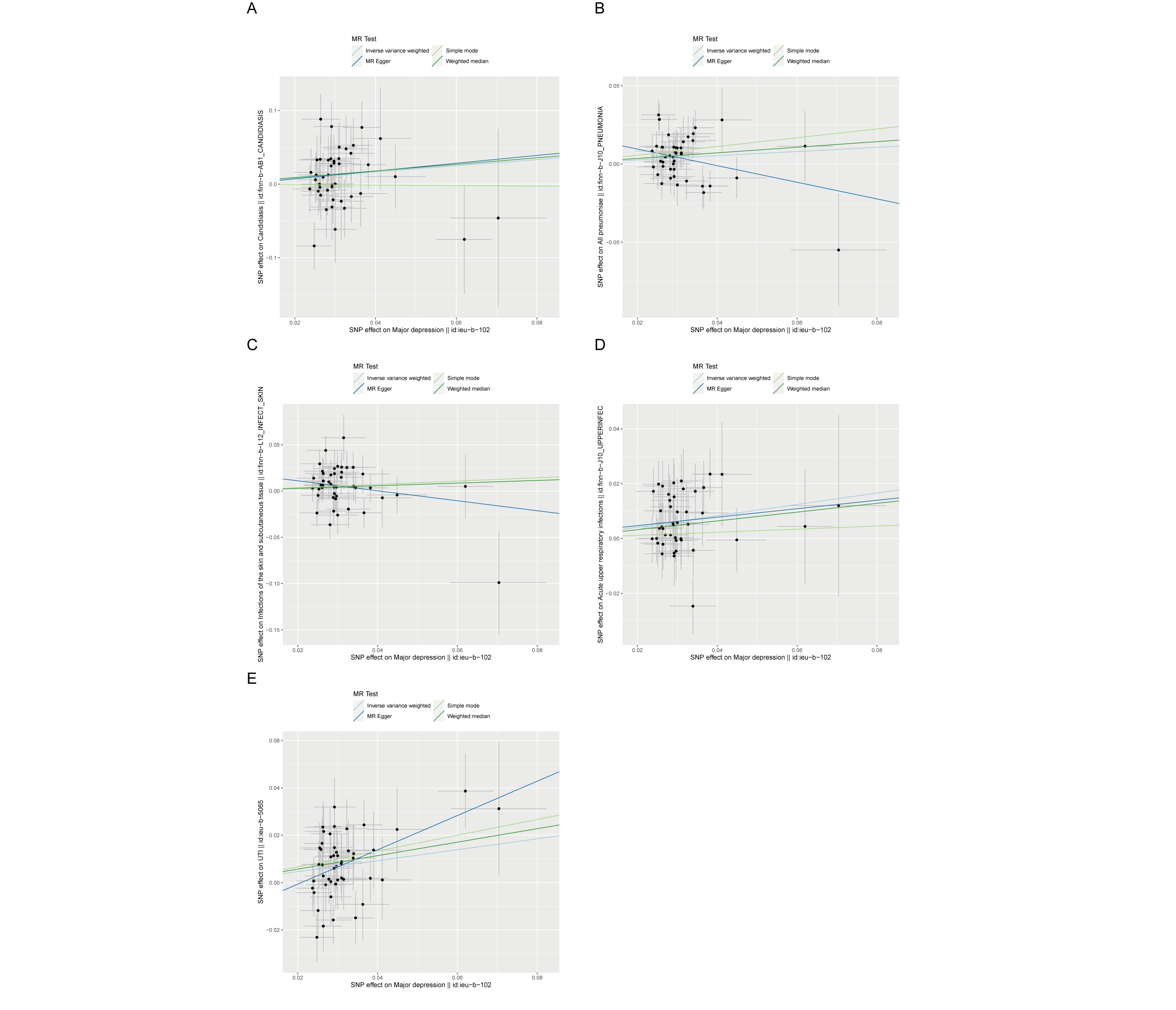

Supplement: Supplementary file 2 — Figure S1 [file 41398_2024_2950_MOESM2_ESM.tif]

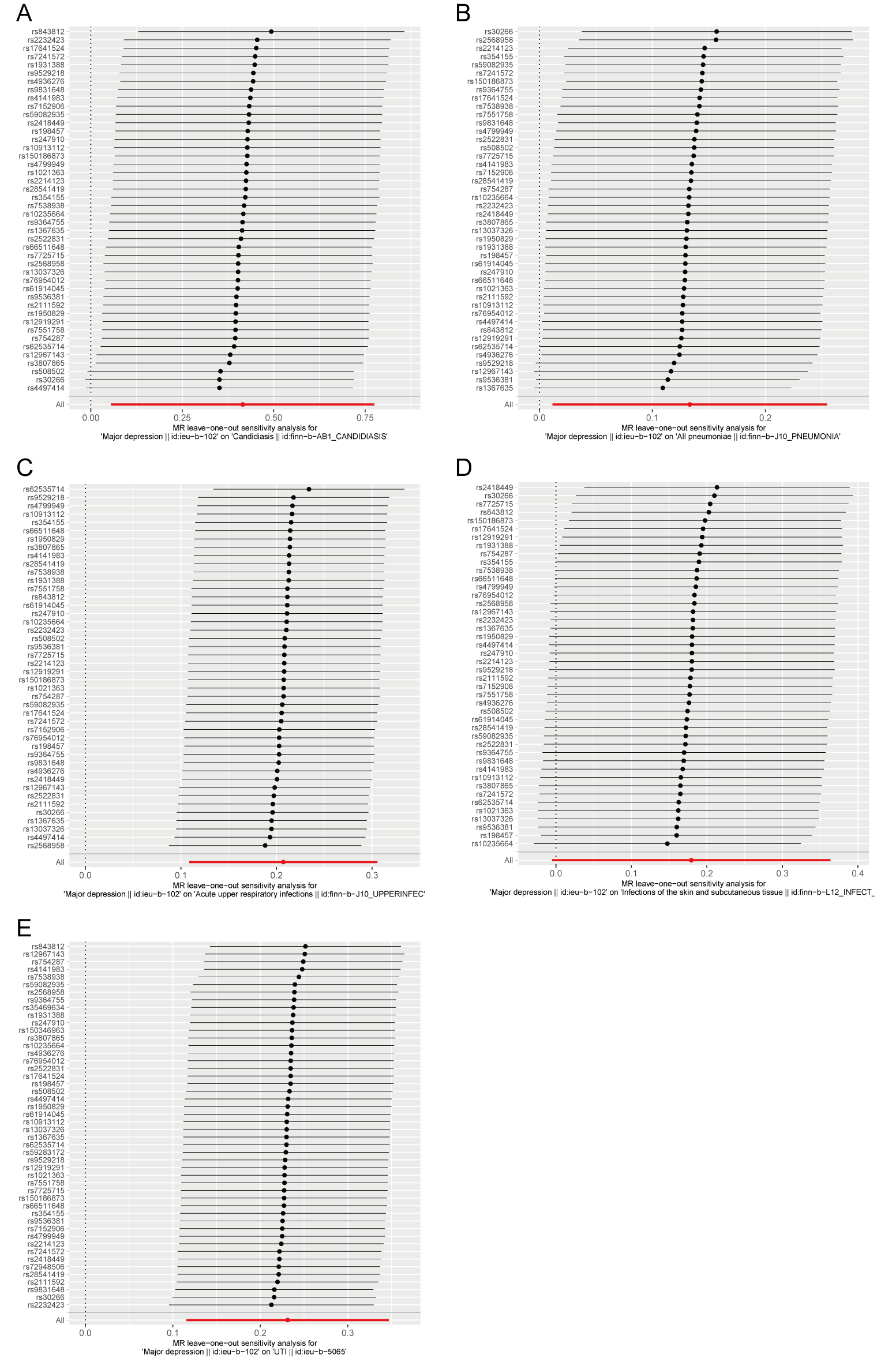

Supplement: Supplementary file 3 — Figure S2 [file 41398_2024_2950_MOESM3_ESM.tif]

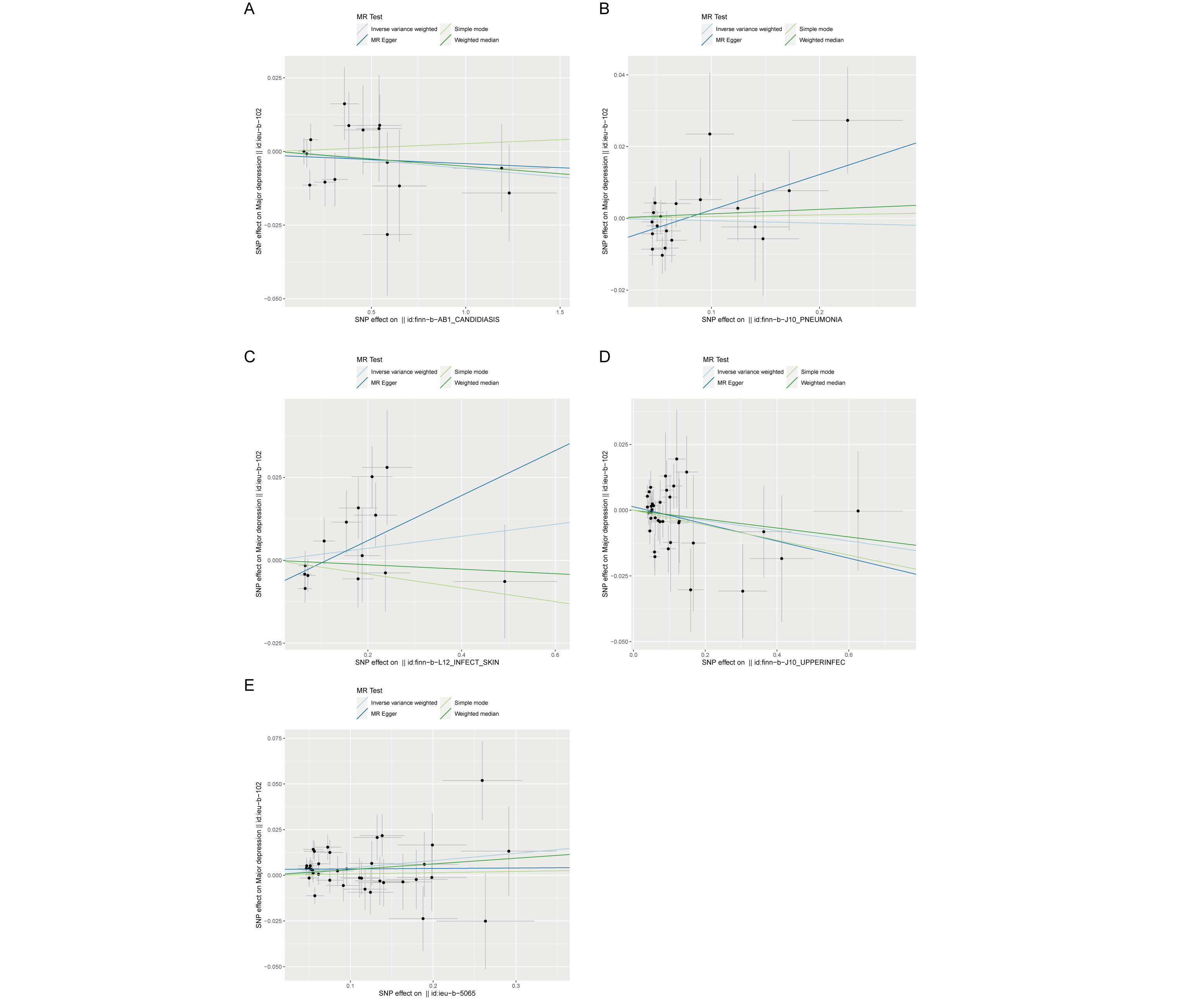

Supplement: Supplementary file 4 — Figure S3 [file 41398_2024_2950_MOESM4_ESM.tif]

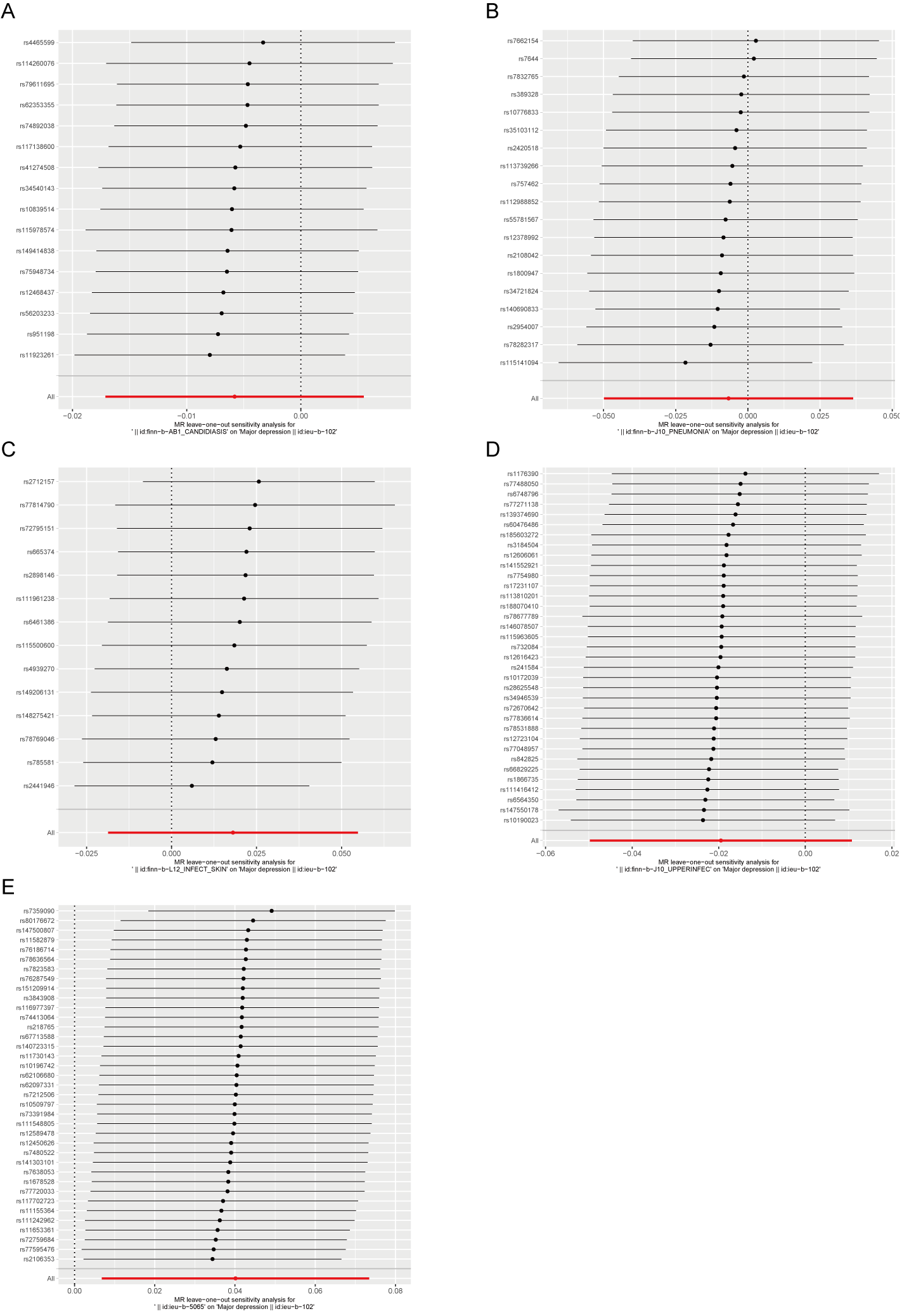

Supplement: Supplementary file 5 — Figure S4 [file 41398_2024_2950_MOESM5_ESM.tif]
